# Some Particulars of a Case of Disease, Exhibiting Phenomena Not Very Dissimilar to Those Evinced in the Yellow Fever of the West Indies, or in That Which Has Been Called the Bulam Fever

**Published:** 1828-08

**Authors:** J. Niell

**Affiliations:** Surgeon H. M. S. Britannia.


### YELLOW FEVER.

The following case was sent to Dr. Burnett, Medical Commissioner of the Navy, by whom it has been forwarded to us.

*Some Particulars of a Case of Disease, exhibiting Phenomena not very dissimilar to those evinced in the Yellow Fever of the West Indies, or in that which has been called the Bulam Fever.* By J. Niell, Esq. Surgeon H. M. S. Britannia.

Thomas Eastly, ætat. forty-nine, messman to the officers of this ship, a man of a sanguine temperament, of a full plethoric habit of body, of a florid, bloated countenance, fond (I believe) of indulging in the pleasures of the table, was seized with strong rigors, at his own house, on the night of the 8th July, succeeded by general uneasiness and a very restless night. The following morning, he complained of a stiffness and soreness over the whole body, as if he had been severely bruised or beaten; of a violent pain in the back, in the course of the spine and loins; headache. Being anxious to return to his duty on board, in attempting to dress himself, the prostration of strength being so great, he fell down in a state of extreme exhaustion. From an impression that it would be of an ephemeral duration, he permitted the disease to continue without molestation till the forenoon of the 11th, when information was received on board of his illness, and that he was unable to get out of bed.

Mr. Crellin, the assistant surgeon, visited him soon afterwards: he found Eastly under considerable febrile excitement; the skin hot; the pulse above 100, full and strong; tongue foul, furred; with constant nausea and vomiting. The patient was immediately bled to syncope, and twenty-eight ounces of blood were abstracted. A cathartic was given, and it operated satisfactorily. At bedtime, Submur. Hydrar. gr. v., Pulv. Antimonial gr. vj. were given in form of pill. The blood did not exhibit any indication of inflammation; it was neither sizy nor cupped; but, on the contrary, the crassamentum was rather convex than otherwise in the centre, with very little serum.

12th.—Had a very restless night. Does not complain of any pain except in the right hypochondrium, in the vicinity of the gall-bladder. Pulse 100, full, soft; skin cool; nausea distressing; retching harassing, aggravated perhaps by the antimonial medicine, and indulging in copious draughts of liquids. He was ordered to take Pulv. Antimon. gr. vj., Submur. Hydrarg. gr. v. every four hours; but, in consequence of the gastric irritability, the antimony was omitted, and the calomel continued. Drinking more than a spoonful of liquid at once was prohibited; and a tablespoonful of Aqua Menthæ was ordered to be taken every hour, with a view to tranquilise the stomach. A blister applied to the right hypochondrium at bedtime.

13th.—Had a very restless night. Side easy; nausea not quite so distressing; thirst urgent; pulse 100; skin cool and soft; the skin of the face and neck of a light yellow tinge; countenance full, flushed, and heavy. Complains of a burning sensation at the pit of the stomach, extending up the œsophagus. The peppermint could not be taken, on account of the pain it excited in the passage to the stomach.

**A dose of the Infusion of Senna and the Carbonate of Magnesia was ordered, and a blister applied to the epigastrium. Submur. Hydrarg. gr. v. were given at bedtime.**

14th.—Had a restless night, but he is free from pain. Nausea still very distressing; vomiting and retching subsided considerably; pulse 100; yellow tinge on the surface is deeper in colour, and extends; the burning sensation at the epigastrium and in the œsophagus is still harassing; singultus; is slightly comatose.

**Ext. Colocynth. comp. gr. ij.; Submur. Hydrarg. gr. v. quartis horis. Effervescing draughts.**

In the evening, pulse about 100, intermitting; some slight mental aberration.

**Omit the Extract of Colocynth, and continue the Calomel gr. v. every two hours. Effervescing draughts. Sherry-wine and brandy ad libitum.**

15th.—Had a very restless night. The nausea has been very distressing; vomited thrice in the night, the matter black like the grounds of coffee. The skin of a deep yellow tinge. The burning sensation at the epigastrium, &c. not quite so distressing. Pulse 120, irregularly intermitting. Singultus troublesome; is slightly comatose; bowels open to purging. Took a bottle of sherry and about half a pint of brandy during the last twenty-four hours, and a teaspoonful of Ether occasionally when the singultus was troublesome. It was consolatory for me to find a highly gifted physician, of great experience both in and out of the tropics, who did me the favor of visiting the patient this day, possess similar diagnostic and therapeutic views on this hopeless occasion with myself; when—

**Calomel gr. x. and Carbonate of Ammonia gr. iv. in pill were to be exhibited every four hours. Wine, brandy, and Ether to be continued as before.**

16th.—Has not had any sleep in the night, but in the morning slept soundly for two hours. He is very easy; the nausea is not so troublesome; the singultus is at times harassing; pulse is not quite so rapid, nor does it intermit so frequently. The yellow tinge on the surface becomes very deep; the face is considerably flushed. The countenance is more animated, and altogether his appearance makes a more favorable impression than hitherto. The bowels are very much relaxed; he has from six to eight evacuations in the twenty-four hours; since the appearance of the black vomit, they (the stools) have likewise been black. The tongue and mouth are covered and lined with a black sordes, parched.

**Wine, brandy, Carbonate of Ammonia, Calomel, and Ether, continued as before.**

17th.—Has been very easy all night, though he has slept but little. The burning sensation in the stomach and œsophagus has disappeared. Pulse about 110, its intermissions are less frequent. Singultus is still very troublesome; nausea less; the alvine dejections watery, but are much lighter in colour, not unlike the washings of a well incrusted port wine-bottle, floating black flaky substances.

Same treatment continued. Sago and strong beef-tea recommended to be given in small quantities, and repeated often.

18th.—Had a very indifferent night; four hours disturbed sleep towards morning. The mouth sore, the gums swelled; mouth and tongue parched, they cannot be kept moist; pulse 100, regular, full, and expanding; bowels open, the evacuations of a dirty brown, floating flaky membranous substances, which I take to be the villous coat of the intestines; singultus and coma less; ecchymosis in various parts of the body.

Treatment the same as yesterday.

19th.—Had a good night, but slept very little. Pulse 96, full, and rather hard; skin soft and pleasant; the yellow suffusion is still deep; the tongue and mouth are much as before; bowels open, the evacuations liquid, the colour of porter, flaky, but in the last some black fecal matter, of a tolerable consistence, was observed. Singultus less; coma more than usual; some reaction towards evening. Takes soup and sago in pretty large quantities, which he retains.

Brandy and part of the wine discontinued. Calomel, Carbonate of Ammonia, and Ether continued. Soup, sago, and beef-tea, as before.

20th.—Is in every respect the same as yesterday.

A bottle of wine in thirty-six hours. Contin[r] alia ut heri.

21st.—Had a tranquil night, but does not sleep. Still comatose. The yellow suffusion on the surface is not quite so deep; the mouth and tongue are still parched, covered and lined with black sordes; gums, cheek, and tongue, sore and ulcerated; bowels open, has from six to ten motions in twenty-four hours: they are from a dark green to a black.

Same treatment continued.

22d.—Though he has passed an easy night, yet he has had no sleep. Singultus has been unusually troublesome; yellow suffusion is not quite so dusky, it is of a bright golden yellow; pulse 100, regular, rather strong and hard; mouth and tongue cannot be kept moist; the mouth very sore, much swelled and ulcerated. Urine passed copiously, of a dark porter-like colour; had about six copious evacuations during the night, the last of a thickish consistence, fecal, of a pale ash colour.

Omit Calomel. Wine a pint in the day. Camphor ℈ss. in die. Æther when singultus is troublesome.

23d.—Had a better night than usual; two hours sound refreshing sleep. Several evacuations in the night, all copious and fœtid, with fecal lumps of a light brown aspect. The mouth very sore and swelled, but it, with the tongue, is kept moist with greater facility than hitherto; the saliva begins to secrete. Pulse about 96; skin of a brighter yellow; singultus less.

**Wine, Camphor, Carbonate of Ammonia, sago, soup, and broth.**

24th.—Is easy. Slept soundly about two hours. Singultus less; pulse 100, full, hard, and strong; the evacuations frequent and copious.

**Wine omitted, except in sago. Camphor, Carbonate of Ammonia, and Æther continued.**

25th.—Had a very good night, but did not sleep more than an hour. Pulse ninety-six, regular, strong, full, and hard; singultus less; tongue and mouth becomes softer and moister; yellow suffusion disappears.

**Continue Carb. Ammoniæ; omit Camphor. Sago, soup as before.**

26th.—Had a better night than usual, slept soundly for several hours. No singultus; pulse much as yesterday; mouth and tongue moist.

**Omit all medicine. Continue sago, soup, &c.**

27th.—Had an indifferent night; was hot, feverish, and restless, which he ascribed to the sultriness of the weather. Pulse ninety, full and strong; skin cool and soft; bowels open; the evacuations of a dark bilious hue; free from singultus; the adnata and skin become of a much brighter colour; ptyalism begins to flow; mouth very sore, much swelled.

**Sago, beef-tea, chicken or mutton broth.**

28th.—Passed a very good night, though the mouth is very sore. The face is much swelled; ptyalism copious. The mercurial fetor is now offensive. Pulse 100, full and strong; bowels open to purging; has no appetite, but takes sago, beef-tea, and mutton-broth plentifully.

29th.—Is much as yesterday. The pulse is rather more frequent. The skin, towards evening, becomes of late hot, and has a slight accession of fever, which I ascribe to the mercurial action. The ptyalism profuse; bowels open, the evacuations generally are feculent. Appetite improves.

30th.—Though the face and mouth are much swelled, yet still his nights are tolerable; ptyalism profuse; pulse much the same; skin soft and pleasant; yellow suffusion disappears; the appetite improves.

**Beef-tea, chicken or mutton broth, in which the crumb of bread is sopped.**

31st.—It will, I presume, be superfluous to trespass this narrative on your time, now the patient is fairly convalescent. Medicine was not given after this period, except small doses of Sulphas Quininæ, which had a good effect in improving the tone of the stomach, and increasing the appetite. It was long before the mouth got well, from the mercury having caused extensive inflammation, and ulceration of the tongue, cheek, and gums.

October 19th.—The strength increases very slowly; the mouth is not yet quite well. The appetite is good, and the bowels are regular; but he suffers from indigestion whenever he commits the least error in diet, and on such occasions he experiences considerable distention and uneasiness of stomach.

In offering to occupy your time with any remarks on this narrative, already too prolix, I must claim the singularity of the case as my best apology.

I may be permitted to observe that, had this case occurred in any of the West Indian Islands, no medical man would have been hardy enough to have pronounced it any thing but a genuine case of Typhus Icterodes, Bulam, or Yellow Fever. In the present instance, there need be no difficulty in admitting this; for the heat and drought had lately been so intense and continued as to give the season a claim to an intertropical character. Under such circumstances, therefore, when fever, marked by a peculiar train of symptoms, occurs in a person of a sanguine temperament, of a gross habit of body, of a bloated countenance, fond of indulging in every comfort his situation could afford, using fatiguing exercise at times in the solar rays, our surprise need not be excited at disease assuming this peculiar type. Fevers of the remittent type, and some bad cases of cholera, were not uncommon in private practice and on shipboard: therefore, under the peculiarities of Eastly's constitution, habits, and occupations, our surprise may vanish when these things are taken into consideration.

It is not the object of the present moment to enter into the various attributes or properties of what is vulgarly called yellow fever. I conceive it will be only necessary for me to endeavour to establish an identity between this and those cases of daily occurrence in the West Indies. It will therefore be requisite to direct the attention to the invasion, progress, and decline of the complaint; and this case will exhibit a train of phenomena and consequences parallel to those in the western endemic. The strong rigors, the great prostration of strength, the deep-seated muscular pains and headache, though common to the first stage of all fevers, proclaim by their severity here a complaint of some peculiar malignity. The second stage comes on with considerable reaction, distressing nausea, vomiting, and retching, which in these fevers are generally conspicuous; and they are soon succeeded by a yellowness of the surface, and an uneasy burning sensation at the epigastrium, extending along the track of the œsophagus. These are invariably present in all intertropical fevers of a malignant type. Consecutively, black vomit appears at no remote period, and this is generally the harbinger of a fatal termination. I have heard, and it is authentically recorded, that patients have recovered from yellow fever in the West Indies after this symptom has appeared; but, in many cases which it has been my lot to see, I have not known a single instance of recovery after this phenomenon. I have been a witness to many cases where the patient had survived this symptom from three to seven days, and in these the alvine secretions bore a strong resemblance to what is noted in the above case. In the few instances recorded of recovery after this symptom had appeared, we observe it has been always marked by changes in the alvine secretions similar to those exhibited in the present case, from a jet black to a dark brown, then gradually becoming lighter as the natural actions and secretions in the intestines were restored. Without any bias or reference to doctrinal points of discussion which have long agitated the medical world with regard to certain properties yellow fever is said to possess, I trust a series of symptoms in this case are detailed impartially,—symptoms which, under similar circumstances, are exhibited in the various stages of the West Indian endemic,—in order to prove an identity. If, on the present occasion, this be admitted, it will prove that this disease is the legitimate offspring of any latitude under certain modifications of season and temperature; and it may likewise disarm us of any fears which we may entertain of its possessing any virulently contagious properties. This has, however, nothing to do with the present subject: the object is to establish, by phenomena exhibited in this case, its identity with the fever which is the terror of Europeans proceeding to our West Indian possessions.

